# Supplementary material for: Polyketide synthase gene clusters in the ericoid mycorrhizal fungus Oidiodendron maius: genome-wide prediction, expression profiling, and functional characterization of knockout mutants
Source: Front Plant Sci. 2026 Sep 16;17:1924430. doi: 10.3389/fpls.2026.1924430 (PMC13623903; doi:10.3389/fpls.2026.1924430)
Supplement: Supplementary file 1 [file SupplementaryFile1.zip › Supplementary Materials/Supplementary Folder 1.zip!/Oidma1_AssemblyScaffolds_Repeatmasked/knownclusterblast/cluster8/jgi.p_Oidma1_37941_mibig_hits.html]

| MIBiG Protein | Description | MIBiG Cluster | MiBiG Product | % ID | % Coverage | BLAST Score | E-value |
| --- | --- | --- | --- | --- | --- | --- | --- |
| AGO59039.1 | PtaJ | BGC0000121 | Pestheic\_acid\_biosynthetic\_gene\_cluster | 39 | 101.627906977 | 317 | 3e-86 |
| EAL89345.1 | conserved\_hypothetical\_protein | BGC0001403 | Trypacidin\_biosynthetic\_gene\_cluster | 39 | 102.790697674 | 298 | 1.1e-80 |
| ADM34146.1 | hypothetical\_protein | BGC0001084 | Notoamide\_/\_stephacidin\_biosynthetic\_gene\_cluster | 36 | 98.3720930233 | 275 | 7.8e-74 |
| CBF90088.1 | conserved\_hypothetical\_protein | BGC0001271 | Emericellin\_biosynthetic\_gene\_cluster | 38 | 97.6744186047 | 270 | 4.3e-72 |
| AAS90039.1 | HypA | BGC0000008 | Aflatoxin\_biosynthetic\_gene\_cluster | 37 | 100.465116279 | 269 | 9.5e-72 |
| BAE71335.1 | hypothetical\_protein | BGC0000004 | Aflatoxin\_biosynthetic\_gene\_cluster | 36 | 100.465116279 | 265 | 1e-70 |
| AAS90113.1 | hypothetical\_protein | BGC0000006 | Aflatoxin\_biosynthetic\_gene\_cluster | 36 | 100.465116279 | 264 | 3e-70 |
| AAS90089.1 | HypA | BGC0000010 | Aflatoxin\_biosynthetic\_gene\_cluster | 36 | 100.465116279 | 262 | 6.8e-70 |
| AAS90020.1 | HypA | BGC0000007 | Aflatoxin\_biosynthetic\_gene\_cluster | 36 | 100.465116279 | 261 | 2e-69 |
| AAS90068.1 | HypA | BGC0000009 | Aflatoxin\_biosynthetic\_gene\_cluster | 36 | 100.465116279 | 258 | 1.7e-68 |
| EAQ84783.1 | hypothetical\_protein | BGC0001219 | Shanorellin\_biosynthetic\_gene\_cluster | 33 | 81.8604651163 | 195 | 1.7e-49 |
